# Biological performance and oviposition preference of tomato pinworm *Tuta absoluta* when offered a range of Solanaceous host plants

**DOI:** 10.1038/s41598-020-80434-7

**Published:** 2021-01-13

**Authors:** Gerson A. Silva, Elenir A. Queiroz, Lucas P. Arcanjo, Mayara C. Lopes, Tamiris A. Araújo, Tarcisio S. V. Galdino, Richard I. Samuels, Nilson Rodrigues-Silva, Marcelo C. Picanço

**Affiliations:** 1 Laboratório de Entomologia e Fitopatologia, CCTA, Universidade Estadual Norte Fluminense Darcy Ribeiro, Avenida Alberto Lamego 2000, Campos dos Goytacazes, Rio de Janeiro CEP 28013-602 Brazil; 2 Departamento de Entomologia, Universidade Federal de Viçosa, Viçosa, Minas Gerais 36570-900 Brazil; 3 Centro de Ciências da Natureza, Universidade Federal de São Carlos, Buri, São Paulo 18290-000 Brazil; 4 Departamento de Fitotecnia, Universidade Federal de Viçosa, Viçosa, Minas Gerais Brazil; 5 Universidade Federal de Sergipe, Nossa Sra. da Glória, Sergipe 49680-000 Brazil

**Keywords:** Invasive species, Agroecology

## Abstract

The tomato pinworm *Tuta absoluta* (Lepidoptera: Gelechuidae) is native to South America and has now become the main tomato pest in Europe, Africa and Asia. The wide range of host plants attacked by this pest has been reported as one of the main reasons for the success of this important insect species. However, the information currently available on the biological performance of *T. absoluta* on Solanaceae has been obtained from a limited number of host species. The Solanaceae family is composed of thousands of species, many of which are potential hosts for *T. absoluta*. Our results showed that the highest oviposition rates occurred on cultivated tomato plants, potato and wild tomato. The lowest rates occurred on “gilo”, “jurubeba”, green pepper and pepper. The highest survival rates of the immature stages occurred on potato and the lowest on pepper, green pepper and “jurubeba”. Female fertility, following infestation of the different plant species, was highest for insects that developed on tomato or potato and the lowest rates were seen on American black nightshade. The net reproductive rate and the intrinsic growth rate were highest on potato and tomato. Cluster analysis grouped tomato and potato as highly susceptible to attack, American black nightshade, juá, eggplant, gilo and wild tomato as moderately susceptible, whilst pepper, green pepper and jurubeba were categorized as resistant to *T. absoluta*. These results clearly demonstrate that the choice of solanaceous host plant species has a direct impact on the fitness parameters of the tomato pinworm as well as survival potential, dispersion and establishment at new sites. These results are important for the planning of integrated pest management strategies.

## Introduction

The introduction of non-indigenous species into new regions can be directly related to natural dispersion or to anthropic actions. Humans have caused accidental and deliberate dispersal of pests for millennia. Ancient human migrations and trade led to the early spread of animals and domesticated plants, along with associated parasites^1–3^. However, the introduction of non-indigenous species has intensified due to increasing global trade, with pest species rapidly spreading around the world, markedly as events post-Columbus^3^. The establishment of nonindigenous species in new regions depends on biotic and abiotic factors. Biotic factors include the presence or absence of natural enemies and the presence of suitable host plants. Abiotic factors are related to suitable environmental conditions and the effectiveness of protective measures adopted by man^4^. In addition, the spread of pest species is correlated with the same ecological or evolutionary phenomena that are responsible for the arrival of nonindigenous species in new regions^5^.

The tomato pinworm *Tuta absoluta* Meyrick (Lepidoptera: Gelechiidae) is currently considered as the most devastating invasive lepidopteran pest of tomato crops worldwide^5–9^. *Tuta absoluta* is native to South America and is a specialist predator of Solanaceae^5,8,10^. The destructive effect of these insects is due to the formation of “mines” within the leaves and the formation of galleries in the stems, fruits and flowers, which can result in losses of up to 100% in tomato crops^11^. *Tuta absoluta* was first reported attacking tomato crops in South America in the 1960s. In Europe, the first detection of *T. absoluta* occurred in Spain in 2006 and during the following years this pest was found in North Africa and sub Saharan Africa. More recently *T. absoluta* was detected in India, causing large losses in tomato crops grown in open fields and in greenhouses^5,7,12–14^. *Tuta absoluta* is now successfully established in Europe, Africa and Asia, becoming the main invasive pest of tomatoes in these regions. Favourable conditions for the establishment of this pest are due of the absence of natural enemies in these recently invaded regions^15,16^ and the abundance of susceptible host plant species^5,10,17,18^.

Tomato, *Solanum lycopersicum* L., is the preferred host when considering *T. absoluta* oviposition and development, but other solanaceous species, cultivated or non-cultivated (wild), have been shown to serve as alternative hosts, especially potato *Solanum tuberosum* L., eggplant *S. melongena* L., tobacco *Nicotiana tabacum* L., wild tomato *Solanum* spp., and thorn apple *Datura ferox* L.^10,19–26^. The Solanaceae are one of the most diverse plant families among the angiosperms, with approximately 9000–10,000 species and approximately 2000 of those species belonging to the genus *Solanum*. Members of this family occur in all the continents, except Antactica. The highest numbers of species are found in tropical and temperate climes, with a significant predominance in the Neotropics. Plants within this family vary from ephemeral herbs to large forest trees and many species are found colonizing areas modified by agricultural practices^27^.

The high diversity and extensive geographic distribution of solanaceous plants may have played an important role in the rapid and continuous spread of *T. absoluta* into new regions. Thus, in this study we seeked to answer the following fundamental question: how is the biological performance of the tomato pinworm influenced by host plant species? To answer this question the oviposition preference of *T. absoluta* on a range of solanaceous species was investigated and biological life tables for the tomato pinworm on each of these plant species were constructed.

## Materials and methods

### Rearing of *Tuta absoluta*

The tomato pinworms used in the bioassays were obtained from a colony which was established from insects originally collected from commercial tomato crops in Viçosa, Minas Gerias State, Brazil in 1994. The colony was initiated with approximately 200 field-collected individuals. The laboratory colony was maintained following the methodology of Silva et al.^28^, and kept under controlled conditions: temperature (25 ± 0.5 °C), photoperiod (12 h L: 12 h D) and relative humidity (75 ± 1%). The insects were reared in wooden cages (40 × 40 × 40 cm) covered with fine mesh netting. One cage was used for oviposition; one for maintaining the leaves with eggs and first instar larvae; one containing second, third and fourth instar larvae and one cage for pupae and adult emergence. The larvae were fed on tomato leaves of the “Santa Clara” variety, cultivated in a greenhouse without application of insecticides. The tomato pinworm population used in the bioassays was approximately the hundredth generation of this insect.

### Plant species used in bioassays

For bioassays, the Solanaceae used here included six cultivated species and four non-cultivated plant species, which occur naturally in tomato growing regions in Brazil. The cultivated solanaceous species were tomato *Solanum lycopersicum* L. (variety: Santa Clara), potato *Solanum tuberosum* L. (variety Ágata), green pepper *Capsicum annuum* L. (variety All Big), pepper *Capsicum chinense* Jacquin (variety Cumari do Pará), gilo *Solanum gilo* Raddi (variety Verde Claro), and eggplant *Solanum melongena* L. (variety Comprida Roxa). The seeds were obtained from a local market. The non-cultivated species were wild tomato *Solanum habrochaites*, American black nightshade *Solanum americanum* Mill., “jurubeba” *Solanum paniculatum* L. and “jua” *Solanum aculeatissimum* Jacq. (1787). The seeds of the above listed species were obtained from a germplasm bank (Vegetable Gene Bank of the Federal University of Viçosa, Minas Gerais, Brazil) or from collecting in vegetable cultivation areas. The plant seedlings were grown in styrofoam cell trays (68 × 34 × 5 cm) with 125 plugs (filled with vermiculite). After 30 days, the seedlings were transplanted to 6 L plastic pots containing substrate composed of 1/3 cattle manure and 2/3 soil. The soil fertility was corrected in accordance with agronomical recommendations. Plants were watered according to the daily necessity of each species. The temperature of the greenhouse was maintained at approximately 27 °C.

### Ethical approval

All international, national and institutional guidelines applicable to the care and use of animals were carefully followed in this study.

## Experiments

### Bioassay of oviposition preference on Solanaceae

Oviposition preference experiments were performed in the greenhouse (27 ± 3 °C; 60 ± 5% RH and 14L:10D) using 12 wooden cages (100 × 100 × 80 cm) covered in netting. One plant of each species was randomly placed in a circle within each cage. Fifty pairs of adult *T. absoluta* (50 two-day-old males and 50 females) were released into each cage and provided with a honey solution (10%) as a food source. Twenty-four hours later the adults were removed and the number of eggs laid on each plant species was counted. Each cage was considered as one replicate. In this experiment 1200 *T. absoluta* adults (600 females and 600 males) and 12 plants of each species were used.

### Mortality and survival of different *Tuta absoluta* developmental stages

This experiment was performed by constructing biological life tables for the tomato pinworm. In the greenhouse, under the same conditions as described above, thirty *T. absoluta* neonate larvae were transferred individually to leaves of each plant species. Prior to starting the experiments all plants were inspected and leaves with any visible symptoms of fungal disease were removed. The larval instars were determined from the width of the head capsule or exuvia from molted larvae. At the end of the fourth instar (prepupae), the larvae were removed and placed in test tubes and fed with pieces of plant leaves from each species until they pupated. The pupae were then sexed and weighed. Mortality of the larvae and pupae were recorded on a daily basis, as was the duration of the larval and pupal stages.

As the adults emerged from the pupae, one male and one female of the same age were transferred to a plastic oviposition cage (11.3 cm in diameter and 23.4 cm in depth). The oviposition cage was closed at the top with fine mesh netting to aid ventilation. In each cage one leaf disc from each host plant (6.2 cm in diameter) was offered for oviposition. The discs were placed in Petri dishes containing a layer of cottonwool moistened with distilled water. The number of eggs laid by the females was recorded daily and the leaf discs were replaced at that time. The adults were fed with a honey solution (10%).

### Data analysis

The oviposition preference data were subjected to analysis of variance (one-way ANOVA; PROC ANOVA; SAS System 2002), and the means were compared using the Scott-Knott test (*p* < 0.05).

The fertility life tables for the tomato pinworm were constructed based on Southwood & Hendersen^29^ and Galdino et al.^8^. The life table parameters were estimated and analysed from the Jack-knife procedure using a program developed by Maia et al.^30^ for SAS (SAS Institute 2000). The Maia method allows for the use of the log-rank test to analyse the different survival curves. The parameters calculated were; net reproductive rate (R_0_), intrinsic rate of natural increase (rm), and finite rate of increase (λ, individuals/females/day), mean generation interval (T), generation doubling time (Dt, day).

The net reproductive rate (R_0_), that is the cumulative summation of lxmx, is age-specific net maternity which is the product of lx (age-specific survival rate) and mx (age-specific fecundity), and was calculated as:

$$R_{0} = \sum\limits_{x = 0}^{\infty } {l_{x} m_{x} }$$

The intrinsic rate of natural increase (rm), where *T* is generation time:

$$r_{m} = \frac{{\ln \left( {R_{0} } \right)}}{T}$$

The finite rate of increase (λ):

$$\lambda = e^{{r_{m} }}$$

The mean generation interval (T):

$$T = \frac{{\log \left( {r_{0} } \right)}}{{r_{m} }}$$

The generation doubling time (Dt):

$$Dt = log_{2} \left( {r_{m} } \right)$$

The oviposition preference was checked for homogeneity of variance (Bartlett test) and normality of errors (Shapiro–Wilk test). As the data was homogenous and normally distributed, no transformation was necessary.

A cluster analysis using the Euclidian distance method was performed on the oviposition preference data, larval mortality, pupal weight, and adult fecundity of *T. absoluta* on the range of Solanaceae used here. The statistical software for this analysis was MINITAB version 17. The figures were created using SigmaPlot 12.5 (Systat Software Inc., San Jose, California, USA) and Inkscape 0.91 (www.inkscape.org).

## Results

**Figure 1:**
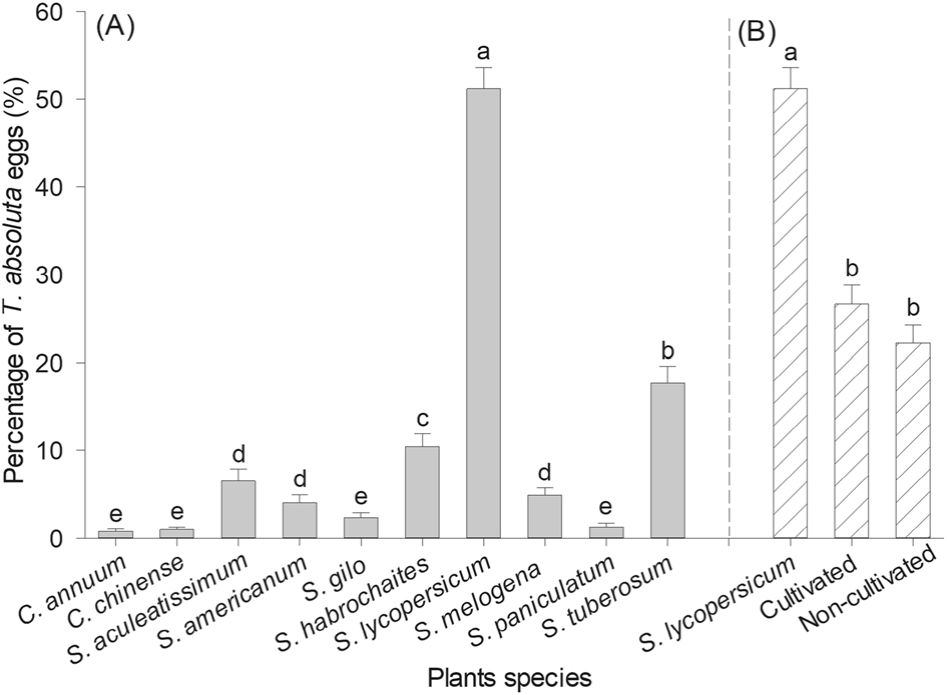
Percentage (mean ± SE) of eggs laid by *T. absoluta* females on leaves of different solanaceous plant species. (**A**) Comparison of all species of solanaceous plants tested here and (**B**) Comparison of tomato to cultivated and non-cultivated solanaceous plants. Means followed by different letters were significantly different according to the Scott Knott test (*p* < 0.05).

*Tuta absoluta* oviposition preference was significantly influenced by the plant species offered to this insect (F = 144.91; df = 9, 110; *p* < 0.001). The highest percentage of eggs was laid on tomato *S. lycopersicum* leaves (51.15%), followed by potato *S. tuberosum* (17.64%) and wild tomato *S. habrochaites* (10.40%) (Fig. 1). Lower percentages of eggs were observed on *S. gilo* (2.32%), *S. paniculatum* (1.27%), *C. chinense* (0.97%) and *C. annuum* (0.79%) (Fig. 1A). There was a significant difference between the percentage of eggs laid on tomato plants when comparing plants grouped into cultivated and non-cultivated solanaceous varieties (F = 48.35; df = 2, 33; *p* < 0.001). Tomato plants were preferred when compared to all other cultivated or non-cultivated plants (Fig. 1B) and the total number of eggs laid on cultivated plant species was similar to the number laid on non-cultivated plants.

**Figure 2:**
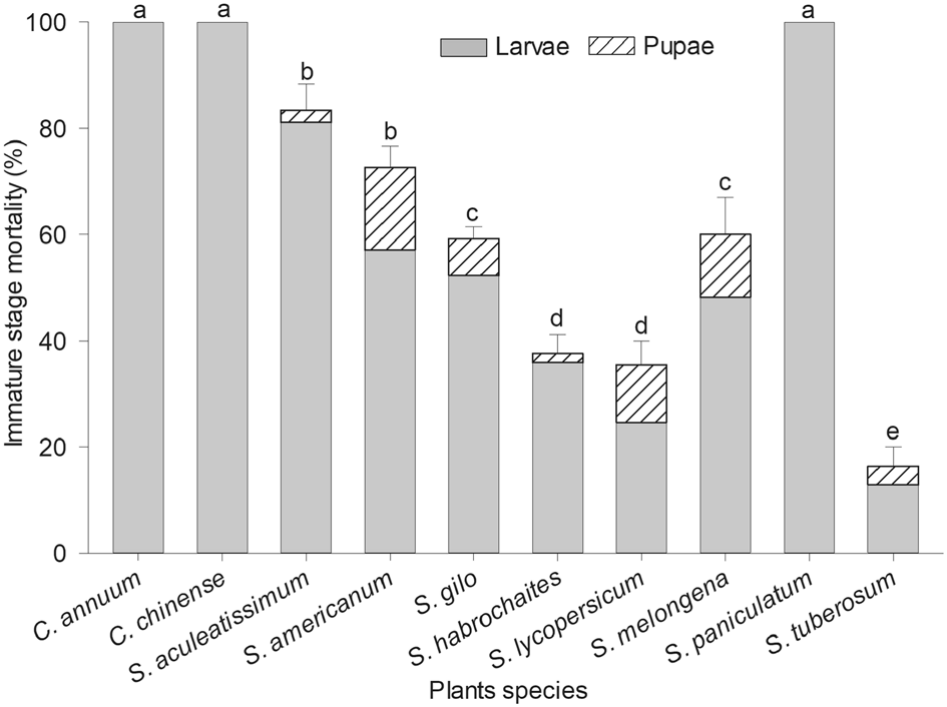
*Tuta absoluta* immature stage (larval + pupal) mortality (mean ± SE) following development on different solanaceous species. Means followed by different letters were significantly different according Scott Knott test (*p* < 0.05).

The mortality rates of the immature stages of *T. absoluta* were significantly affected by the plant species on which they developed (F = 67.80; df = 9, 50; *p* < 0.001). Mortality rates of the larval and pupal stages, when neonate insects were placed on the leaves of different plant species, are shown in Fig. 2. The Scott Knott test separated the plant species into five groups when considering mortality rates (larvae and pupae). The first group consisted of *C. annuum*, *C. chinense* and *S. paniculatum* (100% mortality). The second group consisted of *S. aculeatissimum* (83.33% mortality) and *S. americanum* (72.65% mortality). The third group was *S. gilo* (59.2% mortality) and *S. melogena* (60.1% mortality). The fourth group consisted of *S. habrochaites* (37.55% mortality) and *S. lycopersicum* (35.48% mortality). The lowest rates of larval and pupal mortality occurred on potato plants *S. tuberosum* (16.35%), which was significantly different to the other four groupings (Fig. 2). For *C. annuum*, *C. chinense* and *S. paniculatum*, larvae were observed to continue crawling on the leaf surfaces for long periods without creating mines.

**Figure 3:**
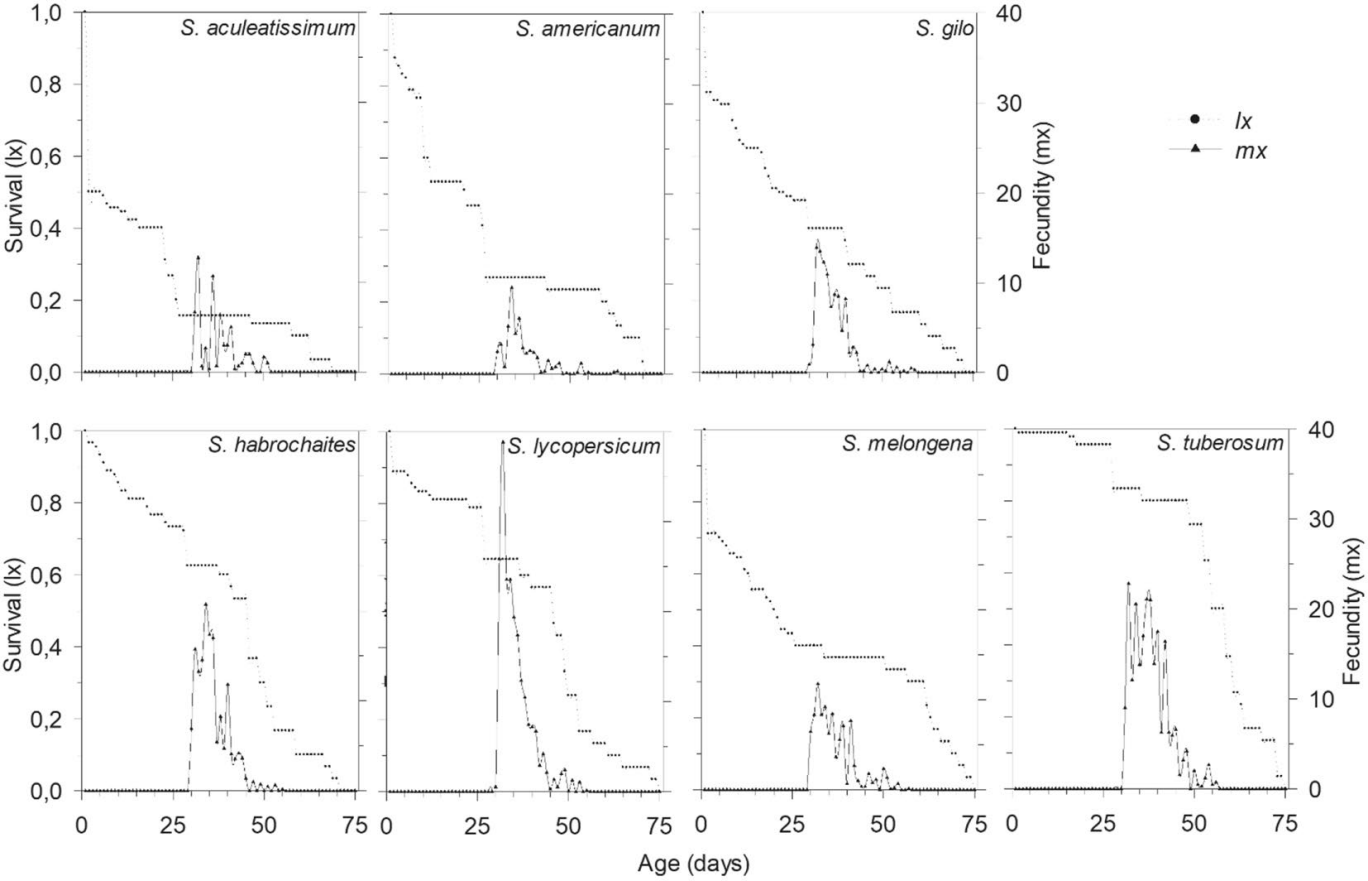
Age-specific survival rates (lx) and fecundity (mx) curves of *T. absoluta* individuals reared on different host plants. Each curve represents the mean values of six cohorts.

In the analysis to determine the effect of the host plant on the biology and fecundity of *T. absoluta*, data for *S. aculeatissimum* was not included due to the low number of adults developing from the immature stages. For *T. absoluta* developmental factors on different host plants, the larval period (F = 50.99; df = 5, 533; *p* < 0.001), pupal period (F = 2.44; df = 5, 533; *p* = 0.034), adult longevity (F = 32.08; df = 5, 152; *p* = 0.009) and lifespan (F = 5.23; df = 5, 152; *p* < 0.001) were all affected by the host plant species. The longest larval period was observed on *S. americanum* (20.47 days) and the shortest on *S. tuberosum* and *S. lycopersicum* (13.86 and 14.52 days respectively; Table 1). *S. americanum* was unique in that development of *T. absoluta* on this plant resulted in an increase in the pupal period (11.58 days). The pupal period was 1.43 days longer than that of insects developing on *S. lycopersicum*. Adult longevity was highest on *S. melogena* and *S. tuberosum* (35.77 and 31.97 days respectively) and lowest on *S. lycopersum* (26.6 days). The shortest lifespan (larval, pupal and adult longevity) was observed on *S. lycopersicum* (53.62 days) but this result did not differ from the lifespan on *S. habrochaites* (51.27 days) and *S. gilo* (54.08 days). On the other hand, the lifespan was highest on *S. melogena* (63.34 days) and *S. americanum* (61.70 days) (Table 1 and Fig. 3).

**Table 1:** Means (± SE) of larval and pupal developmental periods (days), average longevity of males and females (days), and lifespan (days) of *T. absoluta* when offered different solanaceous species.

Plants species	Larval period	Pupal period	Total immature development time	Adult longevity	Lifespan
*S. americanum*	20.47 ± 0.42a	11.58 ± 0.28a	32.05 ± 1.19a	29.65 ± 1.72b	61.70 ± 1.82a
*S. gilo*	15.42 ± 0.29d	10.16 ± 0.25b	25.58 ± 0.49c	28.50 ± 2.55b	54.08 ± 2.53c
*S. habrochaites*	16.37 ± 0.32c	10.52 ± 0.19b	26.89 ± 0.38c	26.73 ± 1.72b	53.62 ± 1.92c
*S. lycopersicum*	14.52 ± 0.30e	10.15 ± 0.39b	24.67 ± 0.72d	26.60 ± 1.75b	51.27 ± 2.13c
*S. melongena*	17.41 ± 0.25b	10.16 ± 0.25b	27.57 ± 0.42b	35.77 ± 1.88a	63.34 ± 1.49a
*S. tuberosum*	13.86 ± 0.17e	10.75 ± 0.21b	24.61 ± 0.35d	31.97 ± 1.57a	56.58 ± 2.25b

Results followed by different letters were significantly different according to the Scott Knott test (*p* < 0.05) when comparing plant species.

Data from *C. annuum*, *C. chinense* and *S. aculeatissinum* were not included in the table because of the high mortality rate of the larvae, making it impossible to continue the life table study of insects on these host plants.

Pupal weight was significantly affected by plant species (F = 32.08; df = 5, 533; *p* < 0.001). The highest weight was observed on *S. americanum* and *S. tuberrosum* (3.63 mg and 3.89 mg respectively). The lowest pupal weight was observed on *S. habrochaites* and *S. melogena* (2.71 mg and 2.56 mg respectively). The pre-oviposition period was also significantly affected by plant species (F = 2.64; df = 5, 72; *p* = 0.03), the longest pre-oviposition period occurred on *S. gilo* (3.50 days), whilst insects offered other plant species had similar pre-oviposition periods. The oviposition periods of adult *T. absoluta* were not affected by host plant species (F = 1.68; df = 5, 71; *p* = 0.15). However, the daily (F = 13.52; df = 5, 72; *p* < 0.001) and total fecundity (F = 12.50; df = 5, 72; *p* < 0.001) were highest when the larvae were reared on *S. lycopersicum*, with a daily mean fecundity of 13.41 eggs/female and a total mean fecundity of 211.88 eggs/female. For potato *S. tuberosum*, daily mean fecundity was 10 eggs/female and total mean fecundity was 188 eggs/female (Table 2). The lowest daily and total fecundities were observed for adults reared from larvae that had fed on *S. americanum* (3.33 eggs/female and 51 eggs/female respectively) (Table 2).

**Table 2:** *Tuta absoluta* mean (± SE) pupal weight (mg), pre-oviposition time (days), oviposition period (days) and fecundity (daily and total) when offered different host plant species.

**Plants species**	**Pupal weight**	**Pre-oviposition time**	**Oviposition period**	**Fecundity (eggs/female)**	
				**Daily**	**Total**
*S. americanum*	3.63 ± 0.13a	2.80 ± 0.36b	15.22 ± 1.64	3.33 ± 0.55c	50.70 ± 8.30c
*S. gilo*	3.13 ± 0.09b	3.50 ± 0.34a	14.42 ± 2.11	6.63 ± 1.51c	95.63 ± 18.09b
*S. habrochaites*	2.71 ± 0.06c	2.53 ± 0.13b	14.06 ± 1.39	9.71 ± 1.35b	136.66 ± 16.32b
*S. lycopersicum*	3.35 ± 0.09b	2.53 ± 0.13b	15.80 ± 1.39	13.41 ± 1.47a	211.88 ± 11.30a
*S. melongena*	2.56 ± 0.08c	2.18 ± 0.18b	19.09 ± 1.35	5.22 ± 0.74c	99.65 ± 14.20b
*S. tuberosum*	3.89 ± 0.09a	3.00 ± 0.40b	18.80 ± 1.24	10.00 ± 1.17b	188.00 ± 23.46a

The values followed by different letters were significantly different according to the Scott Knott test (*p* < 0.05) when comparing different plant species. The total of females tested for the study of *T. absoluta* fecundity were 10 for *S. americanum*, 11 for *S. melongena* and *S. gilo*, and 15 for *S. habrochaites*, *S. lycopersicum* and *S. tuberosum.*

Data for *C. annuum*, *C. chinense* and *S. aculeatissinum* were not included in the table because of the high mortality rate of larvae, making it impossible to continue the life table study of the insects placed on these host plants.

The highest net reproductive rate (R_0_) occurred when insects were reared on potato *S. tuberosum* (81.01) or tomato plants *S. lycopersicum* (72.72). The lower *R*_*0*_ was recorded on *S. americanum* (6.72). The shortest interval mean generation (T) and doubling time (Dt) on tomato *S. lycopersicum* were 30.60 and 4.95 respectively, and the highest T and Dt on *S. americanum* were 37.85 and 13.56. The highest intrinsic rates of natural increase (r_m_) and highest finite rates of increase (λ) were seen on tomato *S. lycopersicum* (0.14 and 1.15 respectively) or for wild tomato *S. tuberossum* (0.13 and 1.14 respectively) (Table 3).

**Table 3:** Biological life table parameters (median ± SE) for *T. absoluta* when developing on different solanaceous species.

Plants species	R_0_	T	r_m_	Dt	λ
*S. americanum* (n = 10)	6.72 ± 1.10d	37.85 ± 0.63a	0.05 ± 0.004d	13.56 ± 1.24a	1.05 ± 0.005d
*S. gilo* (n = 11)	18.23 ± 3.44c	32.17 ± 0.63c	0.09 ± 0.006c	7.59 ± 0.58b	1.09 ± 0.007c
*S. habrochaites* (n = 15)	38.20 ± 4.56b	32.31 ± 0.38c	0.11 ± 0.004b	6.12 ± 0.22c	1.12 ± 0.004b
*S. lycopersicum* (n = 15)	72.72 ± 3.88a	30.60 ± 0.36d	0.14 ± 0.002a	4.95 ± 0.07d	1.15 ± 0.002a
*S. melongena* (n = 11)	18.86 ± 2.69c	34.15 ± 0.58b	0.08 ± 0.004c	8.01 ± 0.39b	1.09 ± 0.004c
*S. tuberosum* (n = 15)	81.01 ± 10.15a	32.73 ± 0.52bc	0.13 ± 0.005a	5.15 ± 0.17d	1.14 ± 0.005a

R_0_ (offspring/individual), T (days), r_m_ (day^−1^), Dt and λ (day^−1^); Values followed by different letters were significantly different of according the Student’s t-test (*p* < 0.05). The total of females tested for the study of *T. absoluta* biological life table parameters were 10 for *S. americanum*, 11 for *S. melongena* and *S. gilo*, and 15 for *S. habrochaites*, *S. lycopersicum* and *S. tuberosum.*

Data for *C. annuum*, *C. chinense* and *S. aculeatissinum* were not included in the table because of the high mortality rate of larvae, making it impossible to continue the life table study of insects placed on these host plants.

**Figure 4:**
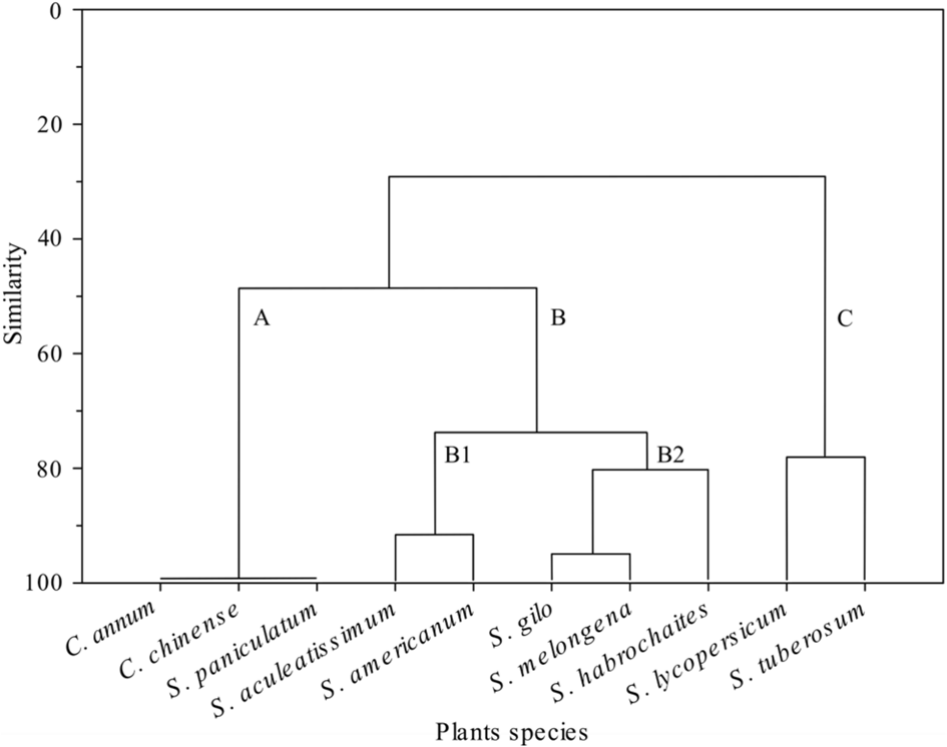
Dendrogram of solanaceous species clustered according to *T. absoluta* performance (oviposition preference, larval mortality, pupal weight, and adult fecundity) when reared on the different plant species.

The cluster analysis (Fig. 4) assembled the plant species into three groups (A, B and C). Cluster A (99% similarity) included *C. annum*, *C. chinense* and *S. paniculatum*, which were classified as unsuitable for *T. absoluta*. Cluster B denominated as the intermediate group, was divided into two subclusters (B1 and B2). Subcluster B1 (92% similarity) grouped *S. aculeatissimum* and *S. americanum* together. Subcluster B2 (80.76% similarity) consisted of *S. gilo*, *S. melongena* and *S. habrochaites*. Cluster C, considered as the most susceptible host plants group (78.40% similarity), consisting of *S. lycopersicum* and *S. tuberosum*.

## Discussion

This is the first study describing oviposition preference and biological performance of the tomato pinworm on cultivated and non-cultivated solanaceous plant species. It was not surprising that adult *T. absoluta* preferred to lay their eggs on domesticated tomato plants (*S. lycopersicum*), with more than fifty percent of the eggs being laid on this species when insects were offered a range of solenaceous plant species at the same time (Fig. 1). This preference for tomato is a result of evolutionary adaptation of *T. absoluta* to tomato toxins and the capacity of females to select sites that are most favourable for the survival of their offspring^8^. Interestingly, the other 50% of the eggs were laid on plants for which the tomato pinworm would not have had any previous contact, especially potato, wild tomato, jua, American black nightshade and eggplant. This demonstrates that *T. absoluta* females can recognize these plants as potential alternative hosts for the development of their offspring. Volatile compounds released by plants play an essential role in the location of suitable host species. These compounds can serve as airborne semiochemicals, promoting or deterring interactions between host plants and insect herbivores^31^. Volatiles from host plants enhance the effect of contact stimulants, increasing landing and oviposition rates when compared to non-host plants, with females capable of detecting small variations in host plant volatile signatures^32,33^.

The high levels of mortality that occurred on *C. annuum, C. chinense* and *S. paniculatum* could be due to the presence of deterrent compounds or physical barriers that prevented larval feeding, causing abandonment of the plants and subsequent mortality due to starvation. Interestingly, the survival rate of the immature stages was higher on potato than on tomato plants. Tomato plants are rich in trichomes, anti-feedant proteins, toxins and protease inhibitors that protect them against pest attack^20,34,35^. The movement of early larval instar *T. absoluta* on the surface of tomato plants is made more difficult because of their small size and the presence of protective barriers (trichomes and hairs) on the leaf surface^36,37^. To overcome these defences, *T. absoluta* larvae bore into the leaves, constructing mines close to the oviposition sites and they remain within the mine, feeding until the third or fourth instar, at which time they have developed morphological and physiological adaptations which enable them to tolerate the tomato defences^8^. This behaviour may be interpreted as an adaptive strategy of the tomato pinworm to avoid host plant defences.

The survival rates and development time of herbivorous insects on different plant species can be considered in the context of life history and adaptation of the insects to host plants^38^. High rates of survival and short development times are indicators of nutritional quality^10,17,18,21,39^. Thus, the higher development times that occurred on American black nightshade could be a reflection of the poor nutritional quality of this host*.* Potato plants could be considered as “better hosts” than tomato plants, as indicated by the higher survival rates, shorter development times and high female fecundity rates. These results are similar to those reported by Megido et al.^17^ when testing four potato varieties, where the development of *T. absoluta* larvae on potato was faster than on tomato plants. Whereas, previous studies^10,18,21,23,25^ found shorter development times on tomato than on potato. One possible explanation for these conflicting results is the range of potato and tomato varieties tested and differences in the genetic make-up of tomato pinworm populations used in each study.

The fecundity of *T. absoluta* was substantially influenced by the solaneceous host plant species. The daily fecundity rate was highest on tomato and lowest on jua, however the total fecundity rate on tomato was similar to that of potato. Fecundity is a good parameter to measure the effect of the host plant on lepidopteran herbivores as the reproductive potential is defined during the larval stage^39,40^. Nutritional quality of the host plant may influence the nutritional reserves and the allocation of resources in larvae, resulting in changes in fecundity, morphology and life history of the insect^41,42^. Previous studies with lepidopterans found a high correlation between body size and fertility, thus a higher immature body mass can result in larger adults, providing greater reproductive success^39,40,43^. This phenomenon occurred in potato, eggplant, tomato and wild tomato, although in gilo and American black nightshade, the higher pupal weight was not reflected in greater fertility of *T. absoluta* adults. Some insect species may compensate for poor nutritional status of the host plant by increasing the consumption rate and protracting larval development. However, these strategies result in a higher risk of juvenile mortality and environmental asynchrony, which may have strong effects on insect population dynamics^40,43,44^.

The net reproductive rate (R_0_) represents the potential of the female offspring to replace the mother throughout her life. This parameter is a good predictor of population growth and is strongly influenced by immature survival rates and female fecundity^44^. The high values of R_0_ calculated for *S. americanum*, *S. gilo*, *S. habrochaites*, *S. lycopersicum*, *S. melongena* and *S. tuberosum* indicated that *T. absoluta* populations would be predicated to increase when larvae developed on these plants. These experiments were performed under controlled conditions, with *T. absoluta* larvae isolated from natural environmental factors (natural enemies and climatic elements). Under field conditions, the natural mortality of *T. absoluta* is around of 92–99%, as verified in ecological life tables by Bacci et al.^16^ and Miranda et al.^36^. The colonization of plants with low nutritional quality may jeopardize the persistence of non-indigenous insects in the environment due the high mortality, decreased fecundity and extended life cycle, increasing exposure to natural mortality factors^15,40,43^. However, environment conditions suitable for survival and the absence of natural enemies may reduce the impact of food quality on the establishment and spread of invasive species into new regions. Cherif et al.^26^ demonstrated that temperature and relative humidity can impact the biological performance of *T. absoluta* on host plants such as tomato, potato and eggplant. On average, 81 females replace the mother when insects were reared on potato, and this number was similar to that on tomato (72.72). A possible explanation for this result could be the low mortality of the immature stages (larvae and pupae) (24%) and high female fecundity (211.88 eggs) following development on tomato and potato plants (13% and 188 eggs). These results are different to those obtained by Pereyra and Sanchez^20^ and Kanle Satishchandra et al.^45^, who documented a higher R_0_ on tomato (49 and 52.15) in relation to potato (14 and 27.60). We attribute these differences to the plant varieties and genetic differences between *T. absoluta* populations used by each research group.

The intrinsic rate of increase (r_m_) denotes the rate at which a population increases in size without resource limitations^46^. The r_m_ is regularly used in entomological research to assess plant antibiosis effects^41,47–50^. A small delay in the reproduction of an organism with a high r_m_ can reduce net reproduction more than proportionally. When the r_m_ is low, fecundity becomes a critical factor in altering the rate of population growth. The r_m_ values for *T. absoluta* were influenced by larval mortality, development time and adult fecundity of insects reared on the range of solanaceous species used here. The highest r_m_ value was recorded for tomato due to higher fecundity and shorter development times (30.6 days) during the immature stages. However, the low r_m_ value recorded on American black nightshade (*S*. *americanum*) was a result of lower fecundity (50.7 eggs/female) and lower immature stage survival rates (72.65%), as well as longer development times (37.85 days).

The dendrogram clusters clearly demonstrated the performance of *T. absoluta* on the host plants tested here. Cluster A plants were considered as unsuitable for oviposition and inhibitory for larval development. Cluster B (both subclusters B1 and B2) was formed of plants that showed moderate resistance to this pest and cluster C was formed of plants that were highly susceptible to *T. absoluta*. Subcluster B2 brought together cultivated plants (gilo and eggplant) and a non-cultivated plant (wild tomato). This cluster received around 18% of the eggs in the oviposition preference test. The immature survival rate was 40–60% and adult fecundity was 89–127 eggs, whilst R_0_ was 18–38, r_m_ 0.08–0.11, giving a biological performance similar or higher than that seen on tomato^17,21,51–53^ and potato^17,21^ in others studies. This finding indicates that these plants may be used as alternative hosts by *T. absoluta*. Cluster C contained only tomato and potato, with a similarity of around 78%. This high similarity was due to the moderate rate of adult oviposition on potato (18% of the eggs during the oviposition preference test), lower larval mortality (20%) and high adult fecundity (188 eggs/female) of insects reared on potato plants. Although potato plants were suitable for *T. absoluta* larval development, there appears to be a barrier that reduces oviposition rates on potato leaves. In wild potato the presence of glandular trichomes (type A and B) are responsible for resistance to oviposition of potato tuber moth *Phthorimaea operculella* (Zeller) (Lepidoptera: Gelechiidae), and this has been related to larval mortality^54,55^. We hypothesize that the presence of trichomes could also be a barrier against *T. absoluta* oviposition on potato, however more studies are necessary to clarify this possibility.

In this study we used a *T. absoluta* population that has been maintained in the laboratory for more than one hundred generations without introducing new individuals. This extended time in the laboratory induces genetic drift due to consanguineous mating, resulting in the production of homozygotic progeny, with a loss of fitness or production of ecotypes unsuitable for field conditions^54,55^. However, when *T. absoluta* adults and larvae were confronted with a range of solanaceous species, they maintained a high capacity to colonize these plants. We believe that field populations of this pest species also possess a very high capacity to colonize a range of solanaceous plants, because of greater genetic diversity resulting from gene flow between populations and highly selective environment pressure^56,57^.

The results clearly demonstrate that *T. absoluta* is capable of laying eggs and developing on a range of cultivated and non-cultivated solanaceous plants under laboratory conditions. In the field and in greenhouses, *T. absoluta* has been reported attacking *S. muricatum* L., *Nicotiana tabacum* L, *S. nigrum* L., *S. eleagnifolium* L., *S. bonariense* L., *S. sisymbriifolium* Lam., *S. saponaceum*, *Lycopersicum puberulum* Ph., *Physalis peruviana*, *Phaseolus vulgaris*, *Lycium* sp. and *Malva* sp^6,19,24,58–61^. However, more studies are necessary to investigate the biological performance of *T. absoluta* on these host plants. It is therefore very important for the effective management of this pest, to eliminate alternative host plants from areas close to susceptible crops and to avoid simultaneous sowing of hosts preferred by *T. absoluta* in adjoining plots.
